# HSP70 and HSP90 Differentially Regulate Translocation of Extracellular Antigen to the Cytosol for Cross-Presentation

**DOI:** 10.1155/2012/745962

**Published:** 2012-09-25

**Authors:** Yu Kato, Chiaki Kajiwara, Ikuo Ishige, Shusaku Mizukami, Chihiro Yamazaki, Shingo Eikawa, Kazuhiro Kakimi, Heiichiro Udono

**Affiliations:** ^1^Laboratory for Immunochaperones, Research Center for Allergy and Immunology (RCAI), RIKEN Yokohama Institute, Yokohama 230-0045, Japan; ^2^BM Equipment CO., ltd., Tokyo 113-0034, Japan; ^3^Department of Immunology, Okayama University Graduate School of Medicine, Dentistry and Pharmaceutical Sciences, Okayama 700-8558, Japan; ^4^Department of Immunotherapeutics, The University of Tokyo Hospital, Hongo 7-3-1, Bunkyo-ku, Tokyo 113-8655, Japan

## Abstract

Antigens (Ag) from cancer or virus-infected cells must be internalized by dendritic cells (DCs) to be presented to CD8^+^ T cells, which eventually differentiate into Ag-specific cytotoxic T lymphocytes (CTLs) that destroy cancer cells and infected cells. This pathway is termed cross-presentation and is also implicated as an essential step in triggering autoimmune diseases such as Type I diabetes. Internalized Ag locates within endosomes, followed by translocation through a putative pore structure spanning endosomal membranes into the cytosol, where it is degraded by the proteasome to generate antigen peptides. During translocation, Ag is believed to be unfolded since the pore size is too narrow to accept native Ag structure. Here, we show that paraformaldehyde-fixed, structurally inflexible Ag is less efficient in cross-presentation because of diminished translocation into the cytosol, supporting the “unfolded Ag” theory. We also show that HSP70 inhibitors block both endogenous and cross-presentation. ImageStream analysis revealed that the inhibition in cross-presentation is not due to blocking of Ag translocation because a HSP70 inhibitor rather facilitates the translocation, which is in marked contrast to the effect of an HSP90 inhibitor that blocks Ag translocation. Our results indicate that Ag translocation to the cytosol in cross-presentation is differentially regulated by HSP70 and HSP90.

## 1. Introduction

The majority of extracellular Ag internalized by DC is processed and presented by MHCII molecules to activate CD4^+^ T cells, but some fraction is integrated into the conventional MHCI antigen-processing pathway to prime CD8^+^ T cells [1, 2]. The latter pathway is termed cross-presentation [3] and is indispensable for elimination of transformed and/or virus-infected cells [4, 5]. In addition to its role in host defense, cross-presentation certainly operates in autoimmunity. Thus, DCs are believed to uptake even self-antigens from cells and organs including mesenchymal tissues, process them, and present antigenic peptides in the context of MHCI molecules to prime self-Ag-specific CD8^+^ T cells. Under certain conditions this can lead to a pathological attack on self-tissues. Several CD8^+^-T-cell-mediated autoimmune diseases have been identified, such as Type I diabetes [6–9], multiple sclerosis [10, 11], nephritis [12, 13], and psoriasis vulgaris [14]. To prevent the development of such diseases, secondary lymphoid organs generally serve as the site for elimination of CD8^+^ T cells recognizing self-Ag with high affinity, a process that has been referred to as cross-tolerance [15–17]. Both cross-priming and cross-tolerance are based on the same cross-presentation mechanism, but the outcome is dictated by many factors in the microenvironment. The details concerning circumstances that maintain or break cross-tolerance were comprehensively discussed recently in an excellent review [18]. 

In cross-presentation, the mechanism by which exogenous Ag translocates from the endosome/phagosome into the cytosol has long been a mystery. However, we recently have shown that cytosolic HSP90 participates in the translocation by pulling Ag out into the cytosol [19, 20]. Available HSP90 inhibitors initially led us to investigate its role in cross-presentation. Importantly, the results elucidated from pharmacological inhibition paralleled those obtained from experiments using mice and cells in which HSP90*α* was genetically ablated [20]. Thus, the use of chemical inhibitors to molecular chaperones such as HSP90 is a reasonable approach to dissect antigen-processing mechanisms. Based on this experience, we tested the effect of HSP70 inhibitors on antigen presentation when they recently became commercially available. We surprisingly found that the HSP70 inhibitor, VER, completely blocked both endogenous and cross-presentation in a dose-dependent manner. Our results presented here together with our previous studies lead to a model in which HSP70 downregulates and HSP90 upregulates Ag translocation during cross-presentation.

## 2. Materials and Methods

### 2.1. Mice

C57BL/6 (B6) mice were purchased from Clea. OT-I (H-2K^b^ restricted, anti-OVA TCR transgenic) mice [21] were kindly provided by Dr. Heath (The Walter and Eliza Hall Institute, Melbourne, VIC, Australia). All mice were maintained under specific pathogen-free conditions in the RIKEN RCAI animal facility according to institutional guidelines. 

### 2.2. Cells

OT-I CD8^+^ CD11b^−^ T cells were purified from splenocytes by depletion of CD11b^+^ cells followed by positive selection of CD8^+^ cells by magnetic separation with the IMag system (BD Biosciences, Franklin Lakes, NJ, USA). The DC2.4 DC cell line was kindly provided by Dr. K. L. Rock (University of Massachusetts Medical School, Worcester, MA, USA).

### 2.3. Antibodies and Reagents

Mouse anti-OVA serum was generated in our laboratory. OVA (Sigma) was used as a model antigen. The proteasome inhibitor MG115 was from Biomol. The HSP70 inhibitors, VER ((5′-O-[(4-cyanophenyl)methyl]-8-[[(3,4-dichlorophenyl)methyl]amino]-adenosine)) and PIF (2-phenylethynesulfonamide), were purchased from TOCRIS bioscience and StressMarq Biosciences Inc., respectively. Endo-Porter was purchased from Gene Tools, LLC. All other reagents including radicicol were purchased from Sigma.

### 2.4. Paraformaldehyde Fixation of OVA

OVA were treated with 2% paraformaldehyde for 24 hours and dialyzed against PBS extensively. The proteins were applied to endotoxin removal PD10 column and used for AF-647 labeling or cross-presentation assay. All procedures were performed in a 4°C cold room.

### 2.5. Antigen Presentation Assay

The direct and cross-presentation assay for OVA is described in our previous reports [19, 20]. To analyze endogenous antigen presentation, plasmid encoding OVA protein (pcDNA3-myc-TEV-flag-OVA) was transfected into DC2.4 cells using a Nucleofector device (Amaxa Biosystems). Transfected cells were incubated with or without chemical inhibitors for 3 hr and then fixed with 0.5% paraformaldehyde (PFA) and 10^4^ cells/well were cultured with 10^5^ cells/well OT-I CD8^+^ T cells. Culture supernatants were collected at the indicated hours of culture and the amount of IFN*γ* released was measured by ELISA. In the cross-presentation assay, DC2.4 cells were pretreated with chemical inhibitors for 15 minutes at 37°C and then pulsed with soluble OVA protein (0.5 mg/mL, unless otherwise indicated) in the presence of inhibitors for 60 min at 37°C. After washing out the free OVA, cells were cultured for another 2 hr in medium for antigen processing and then fixed with 0.5% PFA and quenched with 0.1 M Glycine. Fixed cells were cultured with purified OT-I CD8^+^ T cells in 96-well U-bottom plates (DC 10^4^: T cell 10^5^ cells/well) for the indicated hours. The amount of IFN*γ* released into the culture medium by CD8^+^ T cells was measured by ELISA. 

### 2.6. Membrane Labeling and Uptake of Alexa Flow (AF) 647-OVA for ImageStream

To visualize and quantify the localization of pulsed OVA, the ImageStream system was used in this study following the protocol supplied by the manufacturer. The membranes of DC2.4 were labeled with PKH67 and the cells were then incubated with AF647-OVA (0.5 mg/mL) for 5 min followed by washing three times. The cells were then subjected to ImageStream analysis.

### 2.7. ImageStream Data Acquisition and Analysis

Details can be found in our previous report [20]. PKH67-labeled DCs (2 × 10^6^ cells) were incubated with AF647-OVA (0.5 mg/mL) for 5 min and washed 3 times. The intact, single cells were analyzed by ImageStream. Double-positive cells, consisting of PKH67-labeled DCs and AF647-OVA (R2), were gated in the plots of PKH67 versus AF647 by comparison with the single color plots for each fluorophore (Figures 4(a) and 4(b), left panel). The double-positive cells included two populations: the DCs that had internalized AF647-OVA (R5 and R6) and those to which AF647-OVA was attached on the cell surface and not internalized (R4), as shown in Figures 4(a) and 4(b), right panel. The internalization score is adjusted such that a ratio value of 0.5 (half of the OVA is inside the cell) has a score of zero, so that a ratio value less than 0.5 (more than half of the OVA is outside the cell) has a negative score (R4) and a value greater than 0.5 (more than half is inside) has a positive score (R5 and R6). The phagocytic ratio was calculated by the counts of cells with internalized OVA (R5 + R6) divided by the count of total PKH67-positive DCs (R2 + R3). Gating for colocalized events was based on visual inspection of histogram bins. Representative images of R5 and R6 are shown in Figure 6. R5 was identified as the DC population with a significant proportion of internalized OVA dislocated from the endosomes into the cytosol. R6 was identified as the population in which most of the internalized OVA was still located within the endosomes.

### 2.8. Fluorescence Microscopy for Visualization of Vesicles

DC 2.4 cells, grown for approximately 16 hr on a glass bottom dish, were treated with MG115, with/without 10 *μ*M inhibitors for 15 min. The cell membranes were stained with PKH67 on ice according to the protocol provided by the manufacturer, then pulsed with 0.5 mg/mL AF647-labeled OVA for 5 min at 37°C and fixed. The fluorescent images of the PKH67-labeled DC (green) and AF647-OVA (red) were observed with a KEYENCE BZ-9000 fluorescence microscope using GFP and Cy5 filter sets, respectively. The individual images were overlaid on image-processing software BZ-II analyzer. AF647-OVA, PKH67-DC, and their merged vesicles were defined as red, green, and yellow, respectively, by the software. 

## 3. Results

### 3.1. Less Efficient in Cross-Presentation of Fixed Ovalbumin (OVA)

**Figure 1:**
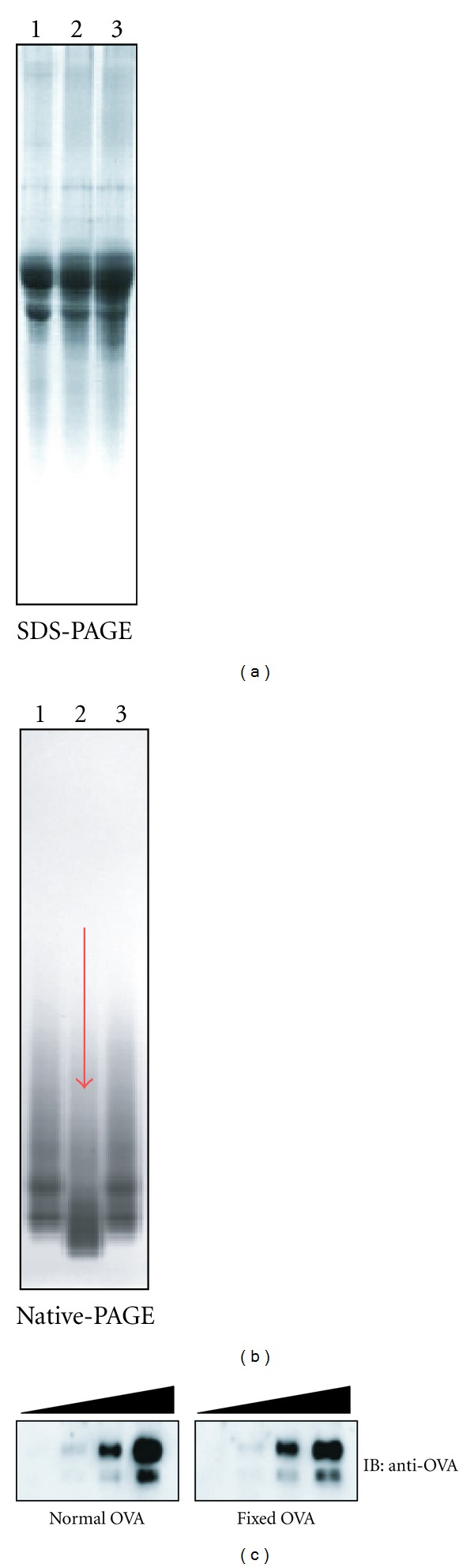
Paraformaldehyde-fixed OVA migrates faster than native OVA in native-PAGE but retains its immunoreactivity. Native OVA (lane 1), paraformaldehyde-fixed OVA dialyzed against PBS (lane 2), and native OVA dialyzed against PBS (lane 3) were analyzed by SDS-PAGE (a) and native-PAGE (b). (c) Native OVA and fixed OVA (1, 0.2, 0.04 *μ*g/lane each) were run on SDS-PAGE, and western blot was performed using anti-OVA polyclonal antibodies.

**Figure 2:**
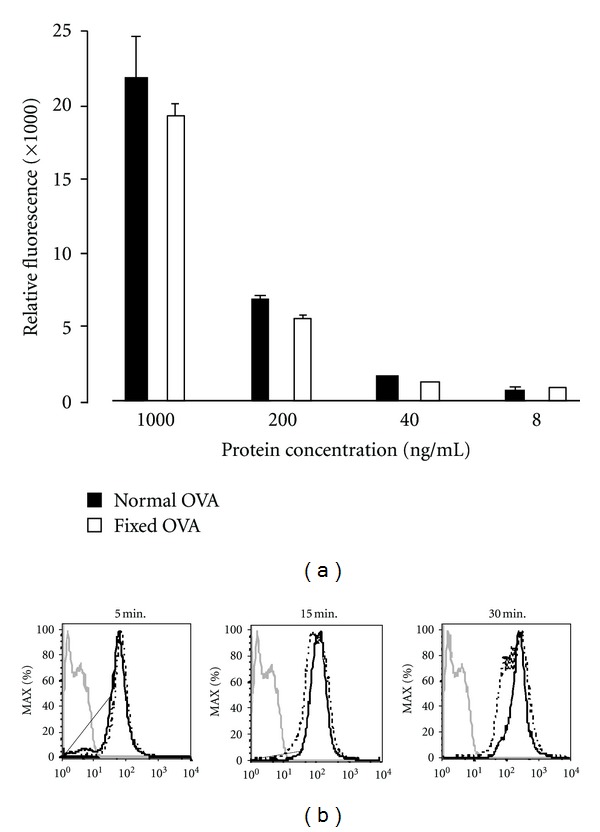
Internalization efficiency by DC2.4 cells is comparable between native and fixed OVA. (a) Labeling-efficiency with AF647 was equal between native and fixed OVA. Native and fixed-OVA were labeled with AF647 and graded doses were analyzed for their relative fluorescence incorporation. (b) DC2.4 cells were incubated with 1 mg/mL AF647-labeled native OVA or fixed OVA for the indicated times and internalization was analyzed by flow cytometer. (- - - -): native OVA, (—): fixed OVA. Gray line: unpulsed DC2.4 cells. Note: MFI = 114 (5 min), 159 (10 min), and 306 (15 min) with normal OVA and 77.3 (5 min), 160 (10 min), and 310 (15 min) with fixed OVA. The results were confirmed in two independent experiments.

**Figure 3:**
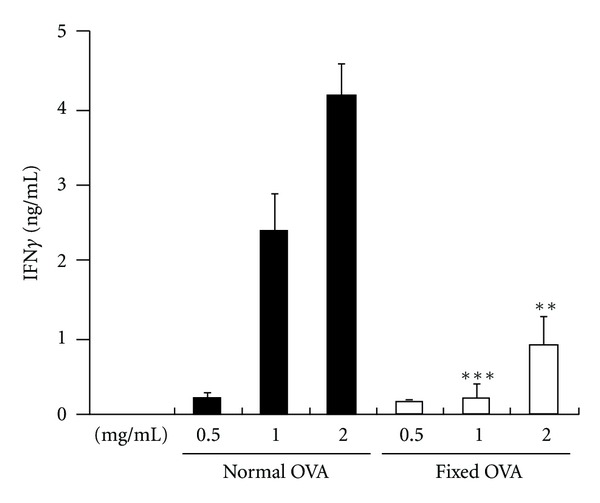
Cross-presentation of fixed OVA is less efficient than native OVA. Graded doses of native and fixed-OVA as indicated were pulsed onto DC2.4 cells for three hours. The cells were then fixed and incubated with OTI-CD8^+^ T cells for 48 hours. The production of secreted IFN*γ* by the T cells was evaluated by ELISA. **P < 0.01; ***P < 0.001. The results were confirmed in at least two independent experiments.

In order to examine nature of Ag required for efficient cross-presentation, we treated OVA with 2% paraformaldehyde for 24 hours and then extensively dialyzed it against PBS. The treated OVA is hereafter referred to as fixed OVA. The fixed or native OVA were indistinguishable by conventional SDS-PAGE analysis (Figure 1(a)). However, fixed OVA were distinguishable by native-PAGE analysis as it migrated significantly faster than native OVA (Figure 1(b), lane 2). Both proteins were detected equally well with antibodies specific to OVA in western blot analysis (Figure 1(c)) and could be labeled to a comparable level with AF647 (Figure 2(a)). To test internalization efficiency, cells of the DC-like cell line DC2.4 were pulsed with AF647-labeled OVA for 5 to 30 minutes. After washing, the cells were analyzed by flow cytometry. As shown in Figure 2(b), internalization efficiency of the two proteins was nearly equal. Based on these results, we proceeded to carry out Ag cross-presentation assays using the fixed and native OVA. Graded doses of both proteins were pulsed onto DC2.4 cells for three hours and unbound OVA was washed off. The DC2.4 cells were then fixed to halt Ag processing, quenched, and incubated with OTI-CD8^+^ T cells (specific to OVA_257-264_-MHCI K^b^ complex) for 48 hours. Ag presentation efficiency was evaluated by quantification of IFN*γ* produced by OTI-CD8^+^ T cells following recognition of MHCI K^b^-OVA_257-264_ complex on cell surface of DC2.4 cells. Notably, the amount of IFN*γ* produced by OTI-CD8^+^ T cells cultured with fixed-OVA pulsed DC2.4 cells was significantly lower than that with native OVA (Figure 3). Thus, Ag presentation efficiency was apparently lower in DC2.4 cells pulsed with fixed OVA than with native OVA. 

### 3.2. Less Efficient Translocation of Fixed OVA from Endosome to Cytosol

**Figure 4:**
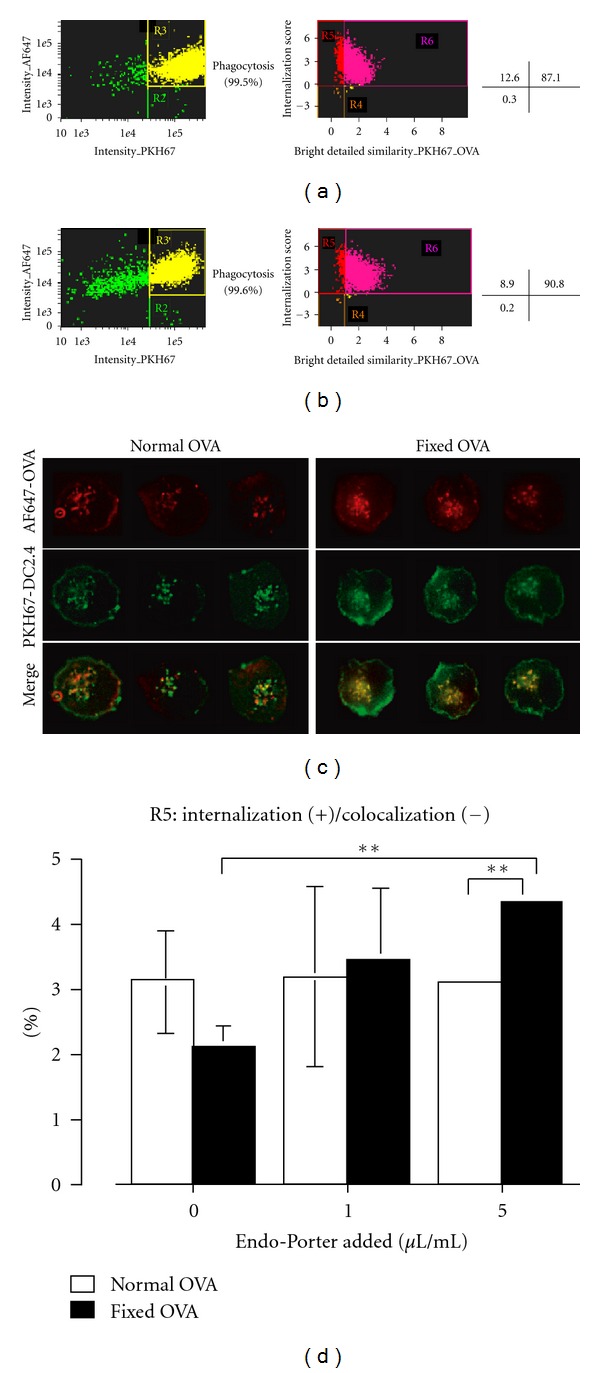
Translocation of fixed-OVA to the cytosol is less efficient than native OVA. 0.5 mg/mL AF647-labeled native OVA (a) and fixed OVA (b) were pulsed onto PKH67-labeled DC2.4 cells for 5 minutes, washed, and subjected to ImageStream analysis. The relative frequency of cells in R5 of native and fixed OVA, thus, internalization (+)/colocalization (−), was 12.6% and 8.9%, respectively. (c) AF647-labeled native or fixed-OVA was pulsed onto PKH67-labeled DC2.4 cells for 5 minutes, washed and subjected to fluorescence microscopy analysis. (d) The same experiments were done as in (a), except that DC2.4 cells were treated with or without Endo-Porter as indicated. The percentage of cells in R5 of native and fixed-OVA were plotted as a bar graph. Note: DC2.4 cells used in ((a)–(c)) were pretreated with 10 *μ*M MG115 15 minutes prior to and during incubation with OVA in order to prevent proteasome activity. Data are mean ± SD of two separate experiments. ∗∗*P* < 0.01.

Since uptake efficiency by DC2.4 cells was equal between native and fixed OVA, we examined translocation of the two proteins following internalization by using the ImageStream system, which can dissect subtle differences in fluorescence distribution within cells and provide a statistical view by analyzing 10,000 cells, as previously we reported [20]. AF647-labeled native or fixed OVA (red fluorescence) was pulsed onto DC2.4 cells whose cell membranes were labeled with PKH67 (green fluorescence). 5 minutes later, the cells were washed and subjected to ImageStream. Intact and single cells were first selected, and then cells double positive for PKH67 and AF647 (identified as cells internalizing OVA, R3 in Figures 4(a) and 4(b), left panels) were further dissected according to bright detailed similarity (BDS) and internalization score. (The detailed principle is described in Section 2.) Populations in R5 and R6 were identified as cells where internalized OVA and membranes were not colocalized and colocalized, respectively. Thus, R5 identified cells where internalized OVA was translocated into the cytosol while R6 identifies cells in which internalized OVA is still retained within the endosome/phagosome. The representative fluorescence images were similar to those in Figure 6(a) (data not shown). Phagocytosis efficiency was comparable, thus, 99.5% and 99.6% with native or fixed OVA, respectively (Figures 4(a) and 4(b)). However, translocation efficiency was lower with fixed OVA (8.9%) compared to native OVA (12.6%), as seen in Figures 4(a) and 4(b), right panel. To further confirm this observation, we used fluorescence microscopy with a focal depth of 0.3~0.4 *μ*m compared to 4 *μ*m in ImageStream. Red fluorescence, representing translocated OVA, was observed much more frequently in cells pulsed with native OVA compared with cells pulsed with fixed OVA (Figure 4(c)). These results indicate that fixed OVA is less efficient in translocation from endosome to the cytosol after internalization. 

The imaging analysis suggests that structural flexibility of OVA is important in translocation; thus, mainly unfolded OVA undergoes translocation. We wondered if a chemical reagent Endo-Porter, which is believed to make transient artificial pores within endosomal membranes so that internalized molecules can be translocated into the cytosol, could affect translocation efficiency following internalization. We treated DC2.4 cells with Endo-Porter during endocytosis and then applied ImageStream analysis. Interestingly, we found that translocation efficiency of fixed OVA was increased by Endo-Porter in dose-dependent manner, whereas that of native OVA was unaffected (Figure 4(d)). 

### 3.3. Differential Inhibition of Endogenous and Cross-Presentation by HSP70 and HSP90 Inhibitors

We previously reported that HSP90 inhibitors completely block cross-presentation but only partially block endogenous presentation and that Ag translocation into the cytosol depends on cytosolic HSP90 [19, 20]. With respect to the cytosolic molecular chaperones, HSC/HSP70 is an abundant heat shock protein, along with HSP90. Since HSC/HSP70 inhibitors became available recently, we investigated their effects on both endogenous and cross-presentation. Among HSP70 inhibitors, VER binds to the ATP binding site of HSC/HSP70 to block its chaperone activity [22–24], while PIF interacts with inducible HSP70 and disrupts its association with cochaperones and substrate proteins [25]. In addition to these two inhibitors, radicicol, an inhibitor to HSP90 was used as a control because we previously used it to dissect the role of HSP90 in cross-presentation [19, 20].

**Figure 5:**
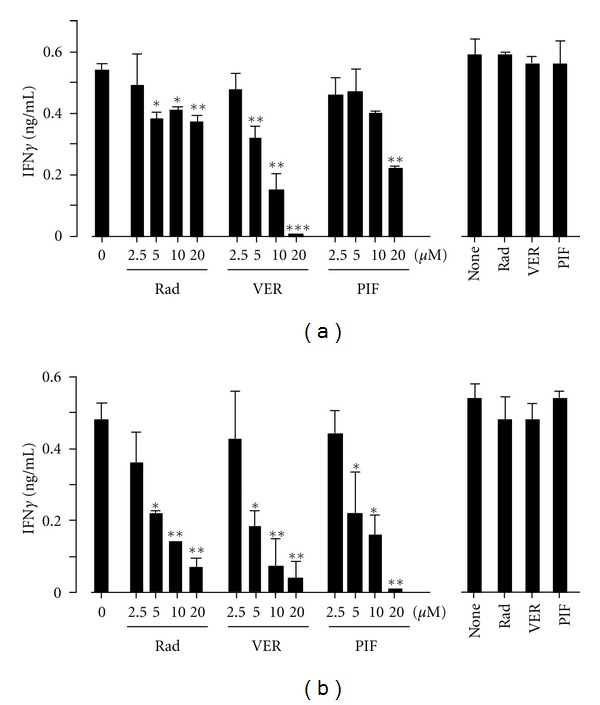
HSP70 inhibitor blocks both endogenous- and cross-presentation. (a) DC2.4 cells were transfected with a plasmid containing cDNA encoding OVA by electroporation and immediately incubated with the indicated inhibitors. Three hours later, the cells were fixed and incubated with OTI-CD8^+^ T cells for 24 hours (left panels). Also, DC2.4 cells transfected with an empty plasmid were pulsed with a suboptimal dose of OVA_(257–264)_ peptide (10^−9^ M) for three hours in the presence of inhibitors (20 *μ*M each), fixed and were used as positive controls in the same assay (right panel). (b) DC2.4 cells were incubated with 0.5 mg/mL OVA in the presence of the indicated inhibitors for three hours and fixed. The cells were washed and incubated with OTI-CD8^+^ T cells for 24 hours (left panel). Also, DC2.4 cells were pulsed with 0.5 mg/mL OVA in the presence of the indicated inhibitors (20 *μ*M each) for three hours, fixed and were used as positive controls in the same assay (right panel). The production of secreted IFN*γ* by T cells in the supernatants was evaluated by ELISA.*P < 0.05; **P < 0.01; ***P < 0.001. The results were confirmed in at least two independent experiments.

Interestingly, we found that VER completely blocked both endogenous presentation and cross-presentation in dose-dependent manner (Figures 5(a) and 5(b), left columns). PIF also blocked cross-presentation completely and showed partial but significant blocking of endogenous presentation (Figures 5(a) and 5(b), left columns). As expected based on our previous results [19, 20], radicicol completely blocked cross-presentation, but there was only partial or marginal inhibition of endogenous presentation (Figures 5(a) and 5(b), left columns). The three inhibitors did not block presentation by DC2.4 cells pulsed with suboptimal dose (10^−9^ M) of OVA_257-264_ peptides (Figures 5(a) and 5(b), right panels).

### 3.4. Differential Effect of HSP90 and HSP70 Inhibitors on Translocation of OVA from Endosome to Cytosol

**Figure 6:**
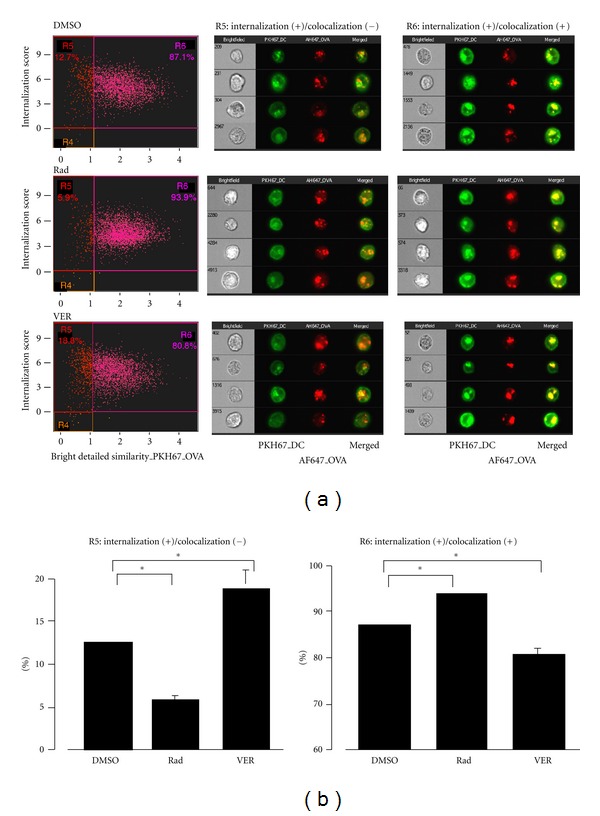
HSP70 inhibitor does not block translocation of OVA to the cytosol following internalization. (a) 0.5 mg/mL AF647-labeled OVA was pulsed onto PKH67-labeled DC2.4 cells for 5 minutes, washed and subjected to ImageStream analysis. DC2.4 cells were treated with the indicated inhibitors (10 *μ*M) 15 minutes prior to and during the five-minute incubation with OVA. The cells were washed and subjected to ImageStream analysis. (b) Data in (a) were plotted as bar graph. Percentage of DC2.4 cells treated with the indicated inhibitors in R5 and R6 are shown. Data are mean ± SD of two separate experiments. *P < 0.05.

The mechanism by which VER blocks cross-presentation was investigated in the context of OVA translocation following internalization by ImageStream. In the solvent (DMSO) control experiment, 12.7% cells were identified as internalization (+)/colocalization (−), thus, translocation (+) (Figure 6(a), upper, left panel, R5). Radicicol, as reported previously [20], downregulated translocation (5.9%) (Figure 6(a), middle, left panel). By contrast, VER did not inhibit translocation but surprisingly increased it (18.8%) (Figure 6(a), bottom, left panel). Fluorescence images of cells treated with each inhibitor (R5 and R6) are shown in Figure 6(a), middle and right panels. Bar graphs summarizing the results of Figure 6(a) are shown in Figure 6(b). These results indicate that an HSP70 inhibitor, VER, rather facilitates or at minimum does not inhibit Ag translocation, which is in marked contrast to the effects of radicicol. 

## 4. Discussion

The present study focused on two important issues—the structural characteristics of Ag required for efficient cross-presentation by DC and the involvement of HSP70 in cross-presentation. These two issues may be seemingly distinct; however, they share a common thread—antigen translocation from endosome to the cytosol.

We showed that paraformaldehyde-fixed OVA was significantly less efficient in translocation from endosome to cytosol following internalization, thus, in cross-presentation, compared to native OVA. As internalization efficiency was nearly comparable between two forms of the protein, the decreased ability of fixed OVA is, at least in part, likely caused by its inflexible structure, which makes it difficult to translocate through a putative translocon in the endosomal membrane. In other words, the pore structure of the translocon is too narrow for native proteins to pass through while keeping their original 3D structure, as previously suggested [1]. Artificial creation of pores in the endosomal membranes with Endo-Porter allowed fixed OVA to translocate to the cytosol, whereas normal OVA translocation was unaffected (Figure 4(d)), suggesting that the translocation mechanism of fixed OVA may be different from that of native OVA. It is likely that translocation of fixed OVA depends simply on the pore size of the translocon. Diffusion to the cytosol in an Ag dose-dependent manner might be a main mechanism operating in this case. By contrast, unfolding of an internalized Ag occurs within the endosome in the case of native OVA, which might provide an opportunity for HSP90 or other molecules to capture and pull the molecule out to the cytosol through the narrow window of the translocon. This concept should be further examined in future's experiments.

We showed that an HSP70 inhibitor, VER, completely blocked cross-presentation as well as endogenous Ag presentation (Figure 5). By contrast, the HSP90 inhibitor, radicicol, blocked cross-presentation completely but showed only partial inhibition of endogenous Ag presentation (Figure 5, [19, 20]). These results suggest that the molecular mechanism inhibited by VER is distinct from that inhibited by radicicol. Radicicol mainly blocks translocation of Ag from endosome to cytosol [20], whereas VER might block a mechanism common to both cross-presentation and endogenous Ag presentation. The common mechanism might be the transport of unfolded Ag to the proteasome. Thus, unfolded Ag emerging across the endosomal membrane or newly synthesized proteins on the ribosome that have become unfolded due to various reasons are captured by HSP70 and forwarded to the proteasome for degradation [26]. Indeed, HSP70 was shown previously associated with Bag1- and HSP70-bound proteins to be degraded are transported to the proteasome [27]. A postproteasomal event might also be a target of VER, since a similar HSP70 inhibitor deoxyspergualin (DSG) blocked MHCI antigen presentation due to dissociation of peptides chaperoned by HSP70 [28]. 

**Figure 7:**
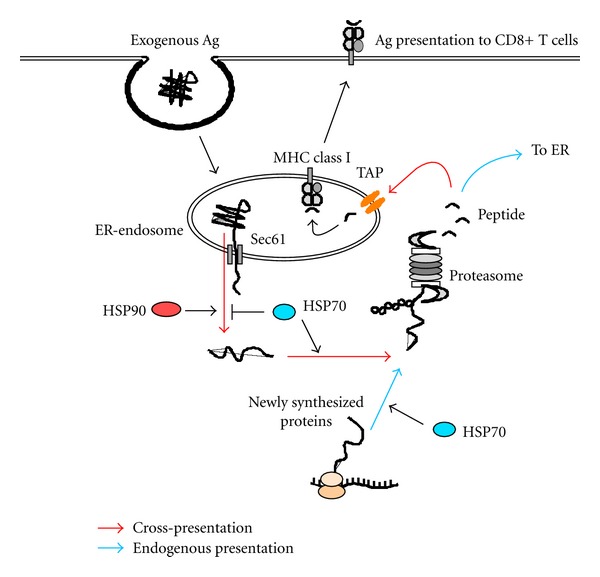
Model illustrating how HSP70 and HSP90 regulate cross-presentation and endogenous Ag presentation. In cross-presentation, internalized exogenous Ag is unfolded within the endosome and translocated through a putative translocon Sec61 complex into the cytosol. The translocation is facilitated by HSP90 but limited by HSP70. The translocated proteins are forwarded to the proteasome for degradation in HSP70-dependent manner. Ag-derived peptides generated by the proteasome enter the same endosome from which it dislocates to the cytosol through TAP molecules and associate with MHCI molecules. TAP molecules and MHCI molecules within the endosome are probably recruited from the endoplasmic reticulum (ER) in a Sec22b-dependent manner. In endogenous Ag presentation, a portion of newly synthesized, unfolded proteins are transported to the proteasome for their degradation by HSP70. Generated peptides enter the ER to associate with MHCI molecules.

In contrast to radicicol, VER facilitates translocation of AF647-labeled OVA to the cytosol, indicating that HSP70 has a suppressive effect on translocation of exogenous Ag to the cytosol. HSP70-dependent, constitutive suppression of Ag translocation would therefore result in downregulation of cross-presentation. This scenario may seem contraindicated in terms of effective host defense. However, blocking of Ag translocation into the cytosol results in Ag retention in endosome, which in turn stimulates fusion with lysosomes, eventually followed by enhanced MHCII antigen presentation. In this context, we recently observed that HSP90*α*-deficient mice showed enhanced T-cell-dependent antibody production [29], whereas T-cell-independent antibody production was unchanged [29]. We believe that this phenotype is caused by a spontaneous increase in the knock-out mice of MHCII presentation because compared to DC of normal mice, splenic DC of HSP90*α*-deficient mice showed increased ability to present Ag to CD4^+^ T cells after pulsing with graded doses of OVA [29]. A model depicting these pathways is shown in Figure 7. 

In conclusion, extracellular Ag to be translocated from endosome to the cytosol needs some structural flexibility, and translocation of Ag to the cytosol is regulated by two distinct cytosolic molecular chaperones, HSP70 and HSP90. The dichotomy of Ag presentation (MHCI versus MHCII presentation of exogenous Ag) regulated by HSP70 and HSP90 might be associated with cross-priming and cross-tolerance, which would give rise to differential outcomes in the onset of autoimmune disease as well as in host defense against cancer and infectious disease.
